# Investigation of cryoprotectants‐treated surimi protein deterioration during chilled and frozen storage: Functional properties and kinetic modeling

**DOI:** 10.1002/fsn3.3510

**Published:** 2023-06-16

**Authors:** Leila Maghsoudi, Marzieh Moosavi‐Nasab, Elahe Abedi, Shahrzad Maleki

**Affiliations:** ^1^ Department of Food Science and Technology, School of Agriculture Shiraz University Shiraz Iran; ^2^ Seafood Processing Research Center, School of Agriculture Shiraz University Shiraz Iran; ^3^ Department of Food Science and Technology, Faculty of Agriculture Fasa University Fasa Iran; ^4^ Department of Civil Engineering, Faculty of Engineering Fasa University Fasa Iran

**Keywords:** chilled and frozen storage, Cryoprotectants, functional properties, modeling studies, surimi protein

## Abstract

The relative cryoprotective effects of flaxseed protein hydrolysate and pectin in comparison with conventional cryoprotectant (sucrose + sorbitol + sodium tripolyphosphates) on stabilization of proteins in surimi of Capoor (*Cyprinus carpio*) were investigated during freezing (−20°C for 4 months) and chilling storage (4°C for 10 days). Although pectin caused to improve water‐holding capacity (27.8%; 4°C and 21.5%; −20°C) on account of highly more inhibitory impact on the ice crystals growth, the protein denaturation may have occurred. It can be related to higher reduction in the amount of salt extractable protein (%) and the immeasurable value of thiol group in surimi formulation containing pectin compared with other cryoprotectants. The results of modeling surimi samples showed that salt extractable protein and sulfhydryl content were in good agreement with the first‐order reaction model at −20°C and second‐order kinetic model at 4°C. In comparison with other samples, samples treated with flaxseed protein showed the lowest reaction rate constant during chilled and frozen storage. The results confirmed that flaxseed protein with no sweetness and considerable caloric value had a cryoprotective effect similar to sucrose + sorbitol + polyphosphate and even better.

## Graphical abstract

The surimi industry demands from numerous white and underutilized species with some of the so‐called “less valuated fish” (Bashir et al., 2017; Martín‐Sánchez et al., 2009). Since great availability, low cost, and good gel‐forming ability of Capoor fish, it was used for surimi production in present study. Although numerous researches have referred the beneficial effect of diverse additives on surimi, the concomitant comparison three categories saccharide, proteinaceous, and hydrocolloid cryoprotectants under chilling and freezing on functional and modeling study during storage time have been not demonstrated. The objectives of the present study are to investigate water binding capacity (%), salt extractable protein (%), and sulfhydryl content of Capoor surimi protein before and after adding different additive categories, namely flaxseed protein hydrolysate (proteinaceous compounds) and pectin (hydrocolloid compounds); to compare their effectiveness with that of the commercial blends (4% sucrose + 4% sorbitol + 0.2% sodium tripolyphosphates); and to study the modeling of protein deterioration during various time of chilling (4°C) and freezing (−20°C).

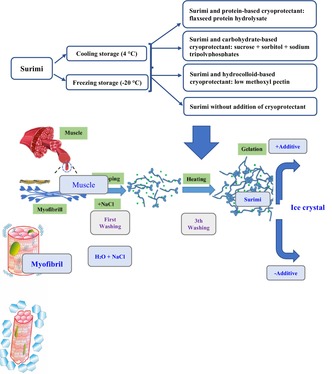

## 1 INTRODUCTION

Surimi, a wet concentrate of proteins, is the deboned, minced, and washed from fish myofibrillar protein, being blended with cryoprotectants to prepare the manufacture of seafood imitation products. Surimi and its derivative are perceived to have wholesome and nutritious attributes (Azadian et al., 2012; Bashir et al., 2017; Martín‐Sánchez et al., 2009).

Although, long‐term storage of marine products is conducted through chilling or freezing, the denaturation of myofibrillar proteins may take place, resulting in loss of functional properties in these proteins (Duangmal & Taluengphol, 2010). The addition of cryoprotectant is required in order to prevent protein denaturation and retain functional properties of protein (Monto et al., 2021). Diverse compounds were found to be used as cryoprotectant including low molecular weight sugars and polyols (sucrose, sorbitol, lactitol, palatinit, and maltodextrin), amino acids, carboxylic acids, and polyphosphates (Cando et al., 2016; Cao et al., 2022; Iglesias‐Otero et al., 2010; Monto et al., 2021) which describe as follow; (1) Saccharide as predominant cryoprotectant is usually consumed to prohibit protein denaturation and degradation of surimi. Moreover, surimi containing saccharide has been exhibited to be capable of maintaining a higher amount of protein than surimi without additives (Arpi et al., 2018). However, this commercial cryoprotectant leads to excessive sweetness and calories. Aside from affecting the taste of the surimi, this restricts the number of consumers on the market, indicating a need to find an alternative (Cao et al., 2022). (2) Protein additives are widely used as proteinase inhibitors in surimi processing to improve the physical properties of surimi gels and to control the activity of heat stable proteinase (Duangmal & Taluengphol, 2010; Monto et al., 2021). Flaxseed protein has no sweetness and contains low caloric value. It contains different levels of polysaccharide gums and emulsion stabilizing compounds which have been assessed as additives in food systems such as canned fish sauce. (3) Hydrocolloids such as carrageenan, pectin, carboxymethylcellulose, and xanthan gums have been utilized as additives in the development of surimi products (Iglesias‐Otero et al., 2010; Monto et al., 2021). Some textural benefits and decrease in production of dimethylamine and formaldehyde were reported for certain hydrocolloids (Martín‐Sánchez et al., 2009).

Various underutilized species are demanded by the surimi industry as “less valued fish” (Bashir et al., 2017; Martín‐Sánchez et al., 2009; Naseri et al., 2013). In the present study, Capoor fish was used for the production of surimi due to its widespread availability, low cost, and good gel‐forming ability. Although numerous researches have referred the beneficial effects of diverse additives on surimi characteristics, the concomitant comparison three categories saccharide, proteinaceous, and hydrocolloid cryoprotectants under chilling and freezing on functional and modeling study during storage time have been not demonstrated. The objectives of the present study are to investigate water binding capacity (%), salt extractable protein (%), and sulfhydryl content of Capoor surimi protein before and after adding different additive categories, namely flaxseed protein hydrolysate (proteinaceous compounds) and pectin (hydrocolloid compounds); to compare their effectiveness with that of the commercial blends (4% sucrose + 4% sorbitol + 0.2% sodium tripolyphosphates). In addition, the protein deteriorations during various time of chilling (4°C) and freezing (−20°C) have been modeled.

## 2 MATERIALS AND METHODS

### 2.1 Materials

Low methoxyl pectin, chlortetracycline, and DTNB (s,s'‐dithio‐bis (2‐nitrobenzoic acid)) were purchased from Sigma company. Other chemical compounds were purchased from Merck Company. Capoor (*Cyprinus carpio*) fish was provided from Fars Fisheries Company of Iran and flaxseed protein hydrolysate with DH 52.0% was provided from Merck Company.

### 2.2 Preparation of surimi

Surimi production was based on the method conducted by Moosavi‐Nasab et al. (2005) with slight modification. Following removing the dark muscle of the filleted Capoor fish, they were cleaned, weighed, and minced. The minced flesh was washed three times with cold water (4°C) for 15 min in each cycle, at a ratio of mince: water 1:4.

**FIGURE 1:**
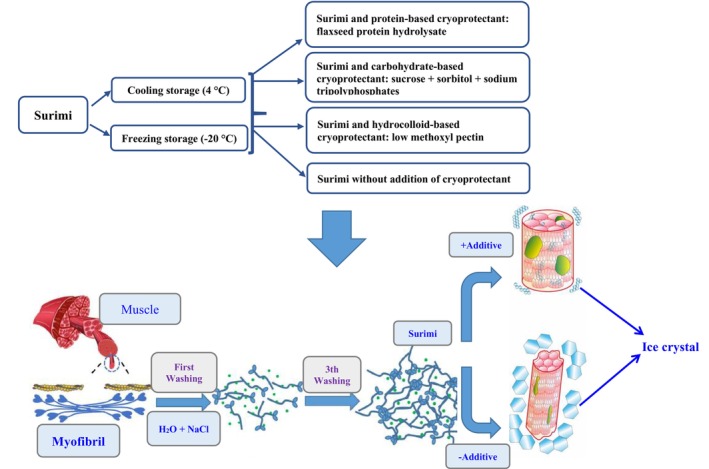
Role of additives in the processing of surimi products under frozen storage.

The first wash water contained 0.2% NaHCO_3_ and 0.1% NaCl to decrease the denaturation rate and increase the sarcoplasmic protein solubility. Due to the removal of hem pigments from the dark‐fleshed fish species (Capoor fish) in alkaline saline solution, surimi becomes lighter in color. The final wash water contained 0.2% NaCl to facilitate dewatering. After each wash, minced flesh dewatered (Figure 1). Final surimi (raw surimi) divided into four portions which are as follow; Group 1 contains surimi without any additives (control sample), Group 2 includes surimi and 4% sucrose + 4% sorbitol + 0.2% sodium tripolyphosphates, Group 3 consists of surimi and 1% flaxseed protein hydrolysate, and Group 4 makes of surimi and 1% pectin.

Surimi was directly incorporated with all additives except pectin by mixing at low‐speed mixer for 1 min. Group 4 was prepared by soaking of surimi (600 g) in LMP solution (1%; 2 L). This slurry was agitated overnight at 4°C and then dewatered.

Finally, four surimi groups are categorized into two groups. One group was stored at −20°C for 4 months, and another group was stored at 4°C for 10 days. Chlortetracycline (5 ppm) and potassium sorbate (1%) was added to the samples to prevent growth of microorganisms under cooling condition (4°C). The frozen and chilled surimi samples were taken for analyses at times 0, 2, and 4 months as well as at times 0, 5, and 10 days, respectively.

### 2.3 Determination of water binding capacity (WBC)

WBC was determined according to Azadian et al. (2012). 5 mL of distilled water was added to 2.5 g of surimi. A slurry was mixed thoroughly and equilibrated at 4°C for 24 h, then centrifuged at 1930 *g* for 10 min at 4°C (Sorvall Centrifuge model MSE). The supernatant was decanted, and the sediment weighted. WBC can be calculated by dividing the weight gain for each pellet by the original weight of each surimi (Azadian et al., 2012).

### 2.4 Determination of salt extractable protein (SEP)

60 mL of NaCl 5% in 40 mM Tris–HCl buffer with pH 7.0 were used to homogenize 15 g of surimi at 3000 *g* for 1 min (Silverson Homogenizer). The homogenate was centrifuged at 9770 *g* for 10 min at 4°C (Sorvall centrifuge model MSE). Afterward, the supernatant was decanted and 60 mL of the same Tris–HCl buffer was added to the pellet and mixture was the centrifuged at 9770 *g* at 4°C for 10 min. The protein content (*N* × 6.25) using micro Kjeldahl method was used to determine the protein concentration in the supernatants. The percentage of protein in the supernatant compared with the total protein concentration before centrifugation is called the SEP% (Sultanbawa & Li‐Chan, 1998).

### 2.5 Preparation of natural actomyosin (NAM)

NAM was prepared as described by Benjakul et al. (1997) with some modifications (Benjakul et al., 1997). 10 g surimi was homogenized (Silverson Homegenizer) in 100 mL of chilled 0.6 M KCl (pH 7.0) for 4 min. The homogenate was centrifuged (Bio‐Dynamic centrifuge) at 8370 *g* for 30 min at 4°C. The precipitated NAM was mixed with three volumes of chilled distilled water. NAM was centrifugated at 8370 *g* (20 min at 4°C), and the pellet was dissolved by stirring in an equal volume of 0.6 M KCl (pH 7.0). By centrifuging at 8370 *g* for 30 min at 4°C, undissolved debris was removed. NAM was maintained in ice during all analyses.

### 2.6 Determination of total sulfhydryl (SH) content

A modified method of Duangmal and Taluengphol (2010) was identified for determining the total sulfhydryl content (Duangmal & Taluengphol, 2010). A 4.5 mL of 0.2 M Tris–HCl buffer solution containing 8 M urea, 2% SDS and 10 mM EDTA (pH 6.8) was added to 0.5 mL of NAM suspension (4 mg/mL). Then, 0.4 mL of 0.1% DTNB in distilled water (pH 7.2) was added to 4 mL of the mixture and incubated at 40°C for 25 min. Using spectrophotometer (model MSE), the absorbance was red at 412 nm. As a blank, 0.6 M KCl was utilized. SH content was determined by the extinction coefficient around 13,600 M^−1^ Cm^−1^.

### 2.7 Determination of drip loss

After 2 and 4 months storage at −20°C, frozen surimi samples were thawed overnight at 4°C, then the drip loss was determined.

### 2.8 Kinetic modeling

According to chemical reactions, the quality of foods changes over time. The kinetic models are used to predict changes in the degradation of food compounds, or the formation of undesired compounds during processing and storage. Depending on the studied reactions, zero‐, first‐, or second‐order kinetic models are used for the food processing (Van Boekel, 1996, 2008).

In order to determine the reaction rate of salt extractable protein and sulfhydryl content changes, the experimental data of surimi samples with different cryoprotectants were averaged and fitted to the first‐ and second‐order kinetic models. The consistency and applicability of these models for predicting data were verified by the coefficient of determination, *R* ^2^, and root mean square error, RMSE. For the first‐order reaction, the equation can be expressed as follows (Van Boekel, 1996, 2008):

(1) $$\frac{\mathrm{dC}}{\mathrm{dt}}=-\mathrm{kC}$$

By integrating both sides of the Equation (1):

(2) $$\ln\frac{C}{C_{0}}=-\mathrm{kt}$$

(3) $$C=C_{0}e^{-\mathrm{kt}}$$

where *C*_0_ is the initial concentration, *C* is the concentration at time *t*, and *k* is the reaction rate constant of the first‐order reaction.

Assuming second‐order kinetic, the rate of reaction can be determined as follows (Van Boekel, 1996, 2008):

(4) $$\frac{\mathrm{dC}}{\mathrm{dt}}=-{\mathrm{kC}}^{2}$$

Integration leads to:

(5) $$\frac{1}{C}=\frac{1}{C_{0}}+\mathrm{kt}$$

(6) $$C=\frac{C_{0}}{1+C_{0}\mathrm{kt}}$$

where *k* is the reaction rate constant of the second‐order reaction.

### 2.9 Statistical analysis

All experiments were carried out in triplicate and the mean and standard deviation were calculated. Duncan's multiple range test using SPSS 22 statistical software (SPSS, Inc.) was implemented to examine the significance of differences between the means (*p* ≤ .05).

## 3 RESULTS AND DISCUSSION

### 3.1 Water binding capacity (WBC) of surimi samples

Surimi gels' WBC is determined by the number of protein–water interactions, which gives an indication of how the water is linked within the protein matrix. WBC of surimi samples containing different cryoprotectants during storage at −20 and 4°C are shown in Tables 1 and 2, respectively. As seen in the Table 1, all ingredients induced an increase in WBC irrespective of the additive after storage under storage at 4 and −20°C. After 4 months storage at −20°C, WBC of the control surimi and surimi mixed with flaxseed protein, sucrose + sorbitol + polyphosphate and pectin, were as 0.36, 0.51, 0.54, and 0.62 (g/g), respectively. WBC decreased as follow, control surimi (58.1%) > surimi + with flaxseed protein (37%) > surimi + sucrose + sorbitol + polyphosphate (30.8%) > surimi + pectin (21.52%).

**TABLE 1:** Water binding capacity (g/g) and salt extractable protein (%), sulfhydryl content (mol/10^5^ g protein) and drip loss (%) of surimi samples with different cryoprotectants during storage (0, 2, and 4 months) at −20°C.

Additives	Water binding capacity (g/g)			Salt extractable protein (%)			Sulfhydryl content (Mol/10^5^ g protein)			Drip loss (%)	
	0 months	2 months	4 months	0 months	2 months	4 months	0 months	2 months	4 months	2 months	4 months
Control	0.86 ± 0.04aA	0.42 ± 0.02bC	0.36 ± 0.04cC	69.8 ± 1.3aA	55.3 ± 0.5bB	46.3 ± 1.5cB	9.4 ± 0.3aA	4.6 ± 0.3bC	4.2 ± 0.2bB	15.0 ± 0.2aA	20.6 ± 0.6bA
Flaxseed protein	0.81 ± 0.04aAB	0.55 ± 0.02bB	0.51 ± 0.04bB	62.8 ± 0.1aC	56.9 ± 1.3bB	55.1 ± 1.5bA	8.7 ± 0.2aB	6.3 ± 0.1bA	5.8 ± 0.4bA	12.3 ± 0.3bB	14.6 ± 0.5aB
Sucrose + sorbitol + polyphosphate	0.78 ± 0.03aB	0.58 ± 0.04bAB	0.54 ± 0.03bB	66.2 ± 1.2aB	63.2 ± 0.7bA	49.4 ± 1.4cB	8.8 ± 0.4aB	5.9 ± 0.2bB	5.5 ± 0.4bA	11.5 ± 0.4aC	12.9 ± 0.7aC
Pectin	0.79 ± 0.03aB	0.64 ± 0.02bA	0.62 ± 0.03bA	22.6 ± 0.8aD	19.3 ± 0.2bC	15.8 ± 0.3cC	ND	ND	ND	9.8 ± 0.5aD	10.5 ± 0.5aD

*Note*: Data fallowed by different small letters in a row show significantly different (*p* < .05) among additives and capital letters in a column are significantly different (*p* < .05) among storage times.

**TABLE 2:** Water binding capacity (g/g) and salt extractable protein (%), sulfhydryl content (mol/10^5^ g protein) and drip loss (%) of surimi samples with different cryoprotectants during storage (0, 5, and 10 days) at 4°C.

Additives	Water binding capacity (g/g)			Salt extractable protein (%)			Sulfhydryl content (Mol/10^5^ g protein)		
	0 days	5 days	10 days	0 days	5 days	10 days	0 days	5 days	10 days
Control with antibiotic	0.87 ± 0.04aA	0.69 ± 0.02bB	0.26 ± 0.03cD	69.1 ± 0.8aA	29.9 ± 1.2bB	27.3 ± 0.4cC	9.3 ± 0.2aA	5.9 ± 0.3bA	4.1 ± 0.3cB
Control without antibiotic	0.86 ± 0.04aA	0.68 ± 0.01bB	0.25 ± 0.02cD	69.6 ± 1.3aA	29.4 ± 0.9bB	26.9 ± 0.9cC	9.4 ± 0.3aA	5.8 ± 0.2bA	4.0 ± 0.5cB
Flaxseed protein	0.81 ± 0.04aAB	0.74 ± 0.02bA	0.39 ± 0.01cB	62.8 ± 0.1aC	48.5 ± 1.5bA	41.0 ± 0.5cA	8.7 ± 0.2aB	5.8 ± 0.1bA	4.6 ± 0.2cA
Sucrose + sorbitol + polyphosphate	0.78 ± 0.03aB	0.69 ± 0.02bB	0.35 ± 0.01bC	66.2 ± 1.2aB	48.3 ± 0.7bA	35.4 ± 0.7cB	8.8 ± 0.2aB	5.4 ± 0.1bB	4.2 ± 0.1cB
Pectin	0.79 ± 0.03aB	0.76 ± 0.03aA	0.57 ± 0.03bA	22.6 ± 0.8aD	16.9 ± 0.2bC	11.2 ± 0.9cD	ND	ND	ND

*Note*: Data fallowed by different small letters in a row show significantly different (*p* < .05) among additives and capital letters in a column are significantly different (*p* < .05) among storage times.

As shown in the Table 2, after 10 days storage at 4°C, WBC of the control surimi containing antibiotic + sorbate (0.26 g/g), control surimi without antibiotic + sorbate (0.25 g/g), surimi mixed with flaxseed protein (0.39 g/g), sucrose + sorbitol + polyphosphate (0.35 g/g) and pectin (0.57 g/g), were determined. As depicted in the results (Table 2), the decrease of WBC in control surimi containing antibiotic + sorbate was not significantly different from control surimi without antibiotic + sorbate. Therefore, antimicrobial agent did not show cryoprotective effect in surimi protein. The reduction percent of WBC was as order, control surimi (70%) > surimi + sucrose + sorbitol + polyphosphate (55.1%) > surimi + flaxseed protein (51.8%) > surimi + pectin (27.8%).

A large number of hydrocolloids from carbohydrates (starch, carrageenan, alginates, xanthan, and high methoxyl pectin) or proteins (fish gelatin, egg white, casein, and beef plasma protein) category have been commonly used as additives in order to improve the mechanical and functional properties (e.g., WBC) of surimi gels (Duangmal & Taluengphol, 2010; Hernández‐Briones et al., 2009), because they are capable of binding with water molecules using various methods. In the case of commercial additive (4% sucrose + 4% sorbitol + 0.2% sodium tripolyphosphates), as phosphate ions are bonded to water, repulsion of protein groups is enhanced due to the predominance of negatively charged protein groups, reducing protein–protein interaction. This allows that more binding sites in protein structures to become available for interaction with water (Cando et al., 2016). On the other hand, in sugar alcohols, more OH groups can contribute to a higher likelihood of interactions with protein and water molecules (Abedi & Pourmohammadi, 2021).

Flaxseed protein contains cystine, lysine and other amino acid residues which promotes SH group protein oxidation to form inter‐molecular disulfide bonds (S–S) and ‐(γ‐glutamyl)lysine cross‐links, respectively (Abedi & Pourmohammadi, 2021; Cando et al., 2016; Pourmohammadi & Abedi, 2021). On the one hand, inter‐molecular disulfide bonds and ε‐(γ‐glutamyl)lysine crosslinks are contributed to construct a gel network structure, improving the gel quality (Duangmal & Taluengphol, 2010). On the other hand, L‐glutamine (L‐Glu) in flaxseed protein hydrolysate cause to increase hydrogen bonds and electrostatic repulsions, modifying the microstructure of surimi, therefore, promoting the WHC of surimi gels (Yuan et al., 2021). By destroying myofibrils, a three‐dimensional gel network is prevented. This action is usually caused by fish's endogenous heat‐activated protease (Duangmal & Taluengphol, 2010). Flaxseed protein hydrolysate possibly possesses as functional binders in surimi gels and also contain protease inhibitors (Udenigwe et al., 2009), resulting in increasing WBC.

Results showed that at both temperatures, WBC in all samples generally decreased during storage; however, this reduction was significantly lower in surimi containing pectin compared with other samples and the highest decrease was found in the control surimi. At the end of storage time at 4°C, decrease of WBC in surimi containing surimi‐added flaxseed protein was significantly lower than sucrose + sorbitol + polyphosphate, while at the end of storage at −20°C, WBC did not show significant difference between sucrose + sorbitol + polyphosphate and flaxseed protein added to as cryoprotectant.

As results conducted by Han et al. (2014), FTIR results showed that three peaks of hydrogen protons can be displayed, implying three water states, including free water, immobilized water, and bound water (Han et al., 2014). Immobilized water, accounting over 95%, was depicted as the main water form in surimi, which are usually generated between myofibrils and had a positive relation with the fish's WHC (Liu et al., 2013). Plus, the amount of bound water covered less than 2%, which is usually tightly bound to macromolecules thus having the lowest fluidity (Zhang & McCarthy, 2012). Meanwhile, free water occupied about 2%, which is easy to lose under the influence of external forces, reducing the surimi's ability to retain water. In consistent with Cao et al. (2022), compared with control surimi, surimi with additives (polyol, flaxseed protein, and pectin) displayed an enhance in the amount of immobilized water and bound water, implying that mentioned additives could limit the flow of water (Cao et al., 2022). Furthermore, it may be identified that due to their interactions with proteins, their structures are stabilized and significantly inhibited the deterioration of protein (Liu et al., 2016). Meanwhile, pectin was seen to employ the most conspicuous restriction on water migration, in turn, enhancing the content of bound water. It suggested that by increasing the viscosity of the composites and forming a network structure using pectin, water flow can be restricted, thereby, preventing ice crystal growth. Cao et al. (2022) examined WHC of surimi gels with different concentrations of inulin (1%, 4%, 8%, and 10% w/w) under varying the number of freeze–thaw cycles. When the amount of additive was augmented to 4% and 8%, the WHC of surimi gels was significantly (*p* < .05) improved, indicating that 4% and 8% inulin + surimi gels exhibited the strongest WHC among repeated freezing and thawing (Cao et al., 2022).

In consistent with present study, Gandotra et al. (2012) and Duarte et al. (2020) noted that fish deterioration under chilled storage (4°C) was more greater than frozen storage (−20°C) which could be attributed to protein denaturation and proteolysis caused by cathepsin, calpain, and collagenase as well as enzymatic activities induced by psychrotrophic microbial growth (Duarte et al., 2020; Gandotra et al., 2012). In addition, produced peptides and free amino acids can promote the microbial growth and production of biogenic amines. In this regard, degradation rate depends on species and storage conditions. The enzymatic actions rate at chilled storage was recorded higher than frozen storage (Duarte et al., 2020).

### 3.2 Salt extractable protein (SEP) of surimi samples

Myofibrillar proteins are soluble in salt solution. Salt solubility is considered as one of the vital characteristics of myofibrillar protein. Cryoprotective additives reduce protein denaturation by preserving salt‐soluble proteins' extractability during cold (4°C) and frozen (−20°C) storage. The extractability of salt‐soluble proteins following the various treatments over 4 months frozen storage and 10 days cold storage are shown in Tables 1 and 2.

As shown in Table 1, during 4 months storage at −20°C, SEP % represented decrease pattern from 69.8% to 46.3% (control surimi) to 62.8%–55.1% (flaxseed protein), 66.2%–49.4% (sucrose + sorbitol + polyphosphate), and 22.6%–15.8% (pectin) after adding diverse additives to surimi. The percent reduction in SEP % of different surimi formulations containing various additives was as follow, control surimi, 33.6%; flaxseed protein, 12.3%; sucrose + sorbitol + polyphosphate, 25.3% and pectin, 30.0%.

As shown in Table 2, during 10 days storage at 4°C, SEP % reduced in all surimi samples. The SEP % reduction of different surimi formulations was as order surimi with or without antibiotic + sorbate (60.4%–61%) > surimi + pectin (50.4%) > surimi + sucrose + sorbitol + polyphosphate (46.5%) > flaxseed protein hydrolyses (41%).

Data analysis depicted that as a function of storage time and temperature, SEP% decreased significantly for control samples (*p* < .05). Generally, the samples' protein solubilities lowered when the storage time increased and the largest percent of the reduction occurred after 2 months (20°C) and 5 days (4°C) of storage time. Concerning to Tables 1 and 2, various additives exhibited diverse inhibitory effect on reducing of SEP%. The SEP% for control surimi decreased swiftly over cold and frozen storage, whereas the SEP% for surimi‐treated flaxseed protein hydrolyses remains relatively stable during this period, implying flaxseed protein hydrolyses protected myofibril proteins from freeze denaturation. Protein denaturation during storage time may be induced by the formation of hydrogen, disulfide or hydrophobic bonds, as well as ionic interactions (Monto et al., 2021; Nopianti et al., 2012), reducing protein solubility (Iglesias‐Otero et al., 2010; Ismail et al., 2012; Li et al., 2014; Nopianti et al., 2012; Zhou et al., 2006). In other word, surimi shows a slower decrease in solubility when a cryoprotectant is added, suggesting cryoprotectants may prevent denaturation of proteins. Ismail et al. (2012) and Nopianti et al. (2012) proved that threadfin bream surimi's protein solubility decreased dramatically. It indicates that proteins were denaturized during frozen storage. Polydextrose, trehalose, and sucrose exhibited a great ability to maintain threadfin bream surimi's protein solubility (Li et al., 2014; Zhou et al., 2006).

In line with Sych et al. (1990), SEP% data for LMP‐ treated surimi was initially low values (22%–15% at –20°C and 22%–11% at 4°C) and also remained low throughout cold and frozen storage. It could be possibly attributed to loss of solubility owing to the incorporation method. Hydrocolloids (pectin, carrageenan, and xanthan) did not display any protective impact on extractable myosin. Although hydrocolloids cause to improve water‐holding capacity on account of the formation of a separate gel in the minced fish matrix, protein denaturation may have occurred during the soaking treatments in hydrocolloids overnight. Thus, their incorporation method is main limiting factor.

### 3.3 Sulfhydryl (SH) content of surimi samples

Among protein functional groups, SH groups are most reactive. SH content of surimi samples with different cryoprotectant during storage at −20 and 4°C are shown in Tables 1 and 2. The SH content of all the samples varied as the function of additive and storage time. As storage time increased, the amount of SH decreased and was significantly different (*p* ˂ .05) from month to month in frozen storage (−20°C) and day to day in cold storage (4°C).

For surimi featuring a cryoprotectant, the SH contents showed the slower trend compared to control surimi during frozen storage. The flaxseed protein hydrolyses‐treated sample showed the lowest decrease around 33% (−20°C) and 47.1% (4°C), followed by sucrose + sorbitol + polyphosphate 37.5% (−20°C) and 52.2% (4°C). A similar decreasing trend in the SH content was also reported by Zhou et al. (2006), Pan et al. (2010) and Nopianti et al. (2012) in tilapia, grass carp surimi and threadfin bream surimi treated with different cryoprotectant during frozen storage, respectively (Nopianti et al., 2012; Pan et al., 2010; Zhou et al., 2006).

When surimi is frozen, ice crystals increase inter‐cellular osmotic pressure, protein molecules are denatured by salting out process. As a consequence, sulfhydryl groups are exposed to oxidation, causing to decrease their contents. Therefore, the amount of total sulfhydryls in a protein is a good indicator of protein oxidation (Cando et al., 2016; Lv et al., 2021; Pan et al., 2010). It is believed that the decrease is caused by the formation of disulfide bonds via the oxidation of SH groups or disulfide interchange, resulting in the aggregation of proteins during freezing or cold storage (Abedi & Pourmohammadi, 2021; Qian et al., 2021). The sulfhydryl oxidation mechanism under chilling storage is somewhat different. The produced free amino acid or small peptide induced by autolysis facilitate to form disulfide linkage during chilling (Duarte et al., 2020). The same trend was observed by Qian et al. (2021) in beef myofibrillar protein after storage at −1 to −18°C for 28 to 168 days (Qian et al., 2021) and Turgut et al. (2016) for refrigerated beef meatballs (Turgut et al., 2016).

At both temperatures of storage, SH content in surimi‐added pectin was not measurable, probably denaturation of the protein may have occurred during the overnight soaking in pectin. The lower rates of denaturation in the other samples suggests that cryoprotection is effective at alleviating denaturation (Nopianti et al., 2012; Pan et al., 2010). The decrease in salt extractable protein is in agreement with the reduce in the total SH level. Moreover, previously Cando et al. (2016) stated improved redisposition of the proteins is rely on the conversion of α‐helical to β‐sheets structures (Cando et al., 2016). The formation of a more ordered network was accompanied with a higher density of cross‐links due to protein aggregation. All in all, in both storage temperatures, flaxseed protein hydrolyses retarded the oxidation of SH groups to disulfide bonds more than any other cryoprotectant. As the result, flaxseed protein depicted the most cryoprotective effect on surimi protein of Capoor, implying it can be an alternative for sucrose + sorbitol + polyphosphate.

### 3.4 Drip loss of surimi samples

Frozen surimi's drip loss is considered a key measure of its quality (Cao et al., 2022). Drip loss of surimi samples with different cryoprotectants after 4 months storage at −20°C are displayed in Table 1. After thawing, water loss rates of all surimi formulations significantly augmented when the time of storage increased (*p* < .05). The drip loss% of the control surimi, surimi containing flaxseed protein, sucrose + sorbitol + polyphosphate, and pectin increased around 37.3%, 15.7%, 10.82%, and 6.6%. As shown in results, at the end of storage at −20°C, drip loss (%) in control surimi was significantly higher than other samples and in pectin‐treated surimi was remarkably lower than other samples.

Possibly there are three reasons for the reduction in water retention capacity of frozen aquatic products: (1) Mechanical damage caused by ice crystal formations or internal stresses on muscle tissue induce to widen cell gaps and to rupture cell membranes. It leads to the loss of extracellular fluid and part of the internal fluid (Lv et al., 2021). The shape and size of ice crystals can also affect the degree of mechanical damage to surimi protein. Therefore, controlling size, shape, and distribution of ice crystals in surimi, positively may cause to prevent freezing damage (Hashimoto et al., 2015; Leygonie et al., 2012). (2) It is possible that the crystallization of water during freezing storage could stretch and squeeze the fish muscle, causing muscle deformations which cannot be completely recovered. Hence, surimi proteins could not re‐absorb water due to the pores left by ice crystals and the muscle damage, enhancing drip loss (Leygonie et al., 2012). Proper cryoprotectants can be used to prevent the formation of large extracellular ice crystals, which would reduce the drip loss. Cao et al. (2022) determined growth of ice crystals in the presence and absence of cryoprotectant. There was significant damage to muscle fibers in the absence of cryoprotectant (Figure 1), because the ice crystals were large and irregular, occupying much of the space and squeezing its structure (Cao et al., 2022). Conversely, the cryoprotectant‐surimi mixtures produced small and regular ice crystals that did not damage the muscle tissue. In numerous studies noted that the water retention capacity of the system will elevate by introducing hydroxyl groups. Inhibiting the ice crystals growth is in additive‐dependent manner. These findings confirmed that the cryopreservation effects of sucrose + sorbitol + polyphosphate and pectin on prevention of drip loss was actually associated to the appearance the hydroxyl groups to a great extent (Leygonie et al., 2012). The inhibition of nucleation and ice crystals growth is ascribed to the restriction influence of additives on free water and a raise in amount of non‐freezing water through enhanced hydrogen bonds, implying that cryoprotectant containing hydroxyl group (sucrose + sorbitol + polyphosphate and pectin) represent a more significant inhibitory impact on the ice crystals growth than flaxseed protein hydrolysate. Moreover, the hydroxyl groups of cryoprotectant embedded into the ice as a result of hydrogen bond interactions may have been resulted in damage to the ice crystals (Zhu et al., 2019). Similar observation was identified by (Cao et al., 2022) after adding different amount of inulin and (Jittinandana et al., 2005) by utilizing of sucrose/sorbitol, trehalose, and trehalose/sorbitol as cryoprotectants. Cao et al. (2022) proved that aside from binding to water, inulin could also interact with myofibrillar proteins via hydroxyl groups and prohibit the aggregation of myofibrillar proteins following freezing, displaying outstanding cryoprotective performance in surimi. As a consequence, a reduction pattern in thawed water loss in reconstructed protein was observed. Similarly, xylooligosaccharides and carrageenan oligosaccharides were found to reduce the area of ice crystals through interaction with the crystals for frozen peeled shrimp, thereby restricting their growth (Zhang et al., 2020). (3) The last major issue is that the protein structural changes decrease their ability to retain water, as a result, the melted water cannot be reunited with the protein molecules and separated from them (Lv et al., 2021). Although pectin represented inhibitory effect on ice crystal growth owing to its hydroxyl groups, regarding the results obtained by SH content and SEP%, protein structure might be denatured which could not re‐absorb thawed water.

### 3.5 Modeling of SH content and SEP of surimi samples

**FIGURE 2:**
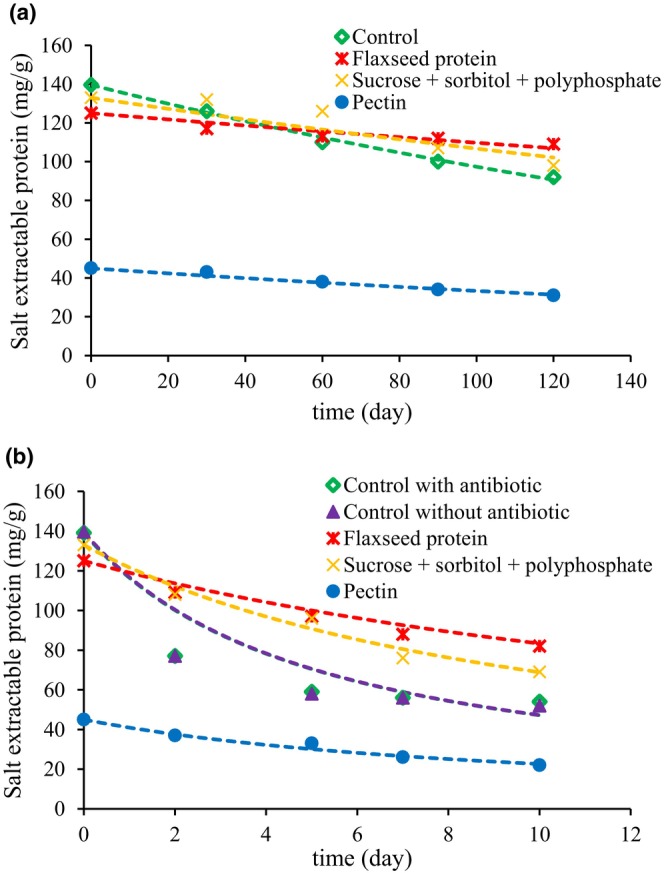
Experimental data and modeling plots of the salt extractable protein changes in surimi samples during storage at (a) −20°C and (b) 4°C.

**FIGURE 3:**
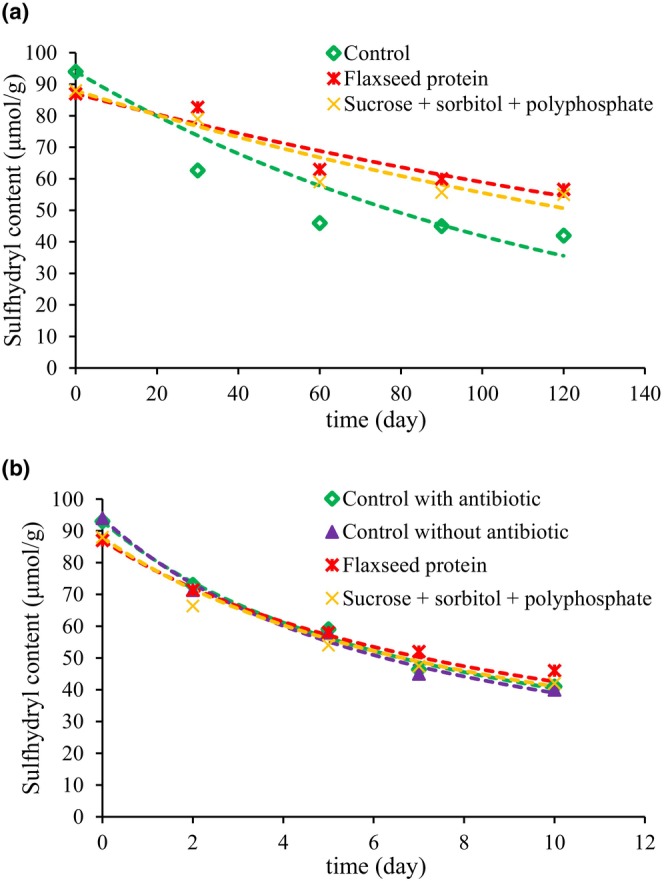
Experimental data and modeling plots of the sulfhydryl content changes in surimi samples during storage at (a) −20°C and (b) 4°C.

Experimental results of changes of the salt extractable protein and sulfhydryl content over time for surimi samples are displayed in Figures 2 and 3, respectively. For all samples, the amounts of salt extractable protein and sulfhydryl content decreased with time.

The data was fitted to first‐ and second‐order reaction models by plotting ln (*C*/*C*_0_) and 1/*C* versus *t*, respectively. The calculated parameters of *k*, *R* ^2^, and RMSE are presented in Tables 3 and 4. According to the *R* ^2^ and RMSE values, the changes of salt extractable protein and sulfhydryl content better fit to the first‐order kinetic model at −20°C, while the second‐order kinetic model is in better agreement with the changes of salt extractable protein and sulfhydryl content values at 4°C. The results of fitting the data to the first‐order kinetic equation at −20°C and second‐order kinetic equation at 4°C are also shown in Figures 2a and 3a and Figures 2b and 3b, respectively. According to Tables 3 and 4, the reaction rate constants of first and second‐order kinetics, *k*, in samples stored at −20°C were much smaller than similar samples stored at 4°C, indicating that storage at −20°C decreased the rate of protein denaturation in samples. As the amount of salt extractable protein and sulfhydryl content in different surimi samples after 4 months frozen storage were higher than similar samples stored for 10 days at 4°C.

**TABLE 3:** Kinetic parameters for salt extractable protein changes in surimi samples during storage at −20 and 4°C.

Additives	Temperature (°C)	First order kinetic			Second order kinetic		
		*k* (day)	*R* ^2^	RMSE	*k* (g/mg/day)	*R* ^2^	RMSE
Control with antibiotic	−20	‐	‐		‐	‐	
	4	0.1197	0.702	17.526	0.0014	0.860	12.002
Control without antibiotic	−20	0.0036	0.994	1.374	0.00003	0.994	1.351
	4	0.1228	0.723	17.193	0.0014	0.862	12.134
Flaxseed protein	−20	0.0013	0.855	2.102	0.00001	0.849	2.149
	4	0.0462	0.961	3.039	0.0004	0.955	3.267
Sucrose + sorbitol + polyphosphate	−20	0.0022	0.833	5.780	0.00002	0.786	6.540
	4	0.0704	0.962	4.489	0.0007	0.975	3.605
Pectin	−20	0.0030	0.971	0.890	0.00008	0.951	1.171
	4	0.0727	0.976	1.246	0.0022	0.971	1.374

**TABLE 4:** Kinetic parameters for sulfhydryl content changes in surimi samples during storage at −20 and 4°C.

Additives	Temperature (°C)	First order kinetic			Second order kinetic		
		*k* (day)	*R* ^2^	RMSE	*k* (g/mg/day)	*R* ^2^	RMSE
Control with antibiotic	−20	‐	‐	‐	‐	‐	‐
	4	0.0889	0.975	2.981	0.0014	0.992	1.643
Control without antibiotic	−20	0.0081	0.839	7.790	0.0001	0.813	8.393
	4	0.0936	0.964	3.714	0.0015	0.990	1.924
Flaxseed protein	−20	0.0039	0.912	3.697	0.00006	0.901	3.918
	4	0.0697	0.963	2.813	0.0012	0.985	1.785
Sucrose + sorbitol + polyphosphate	−20	0.0046	0.901	4.265	0.00007	0.924	3.747
	4	0.0828	0.924	4.501	0.0013	0.974	2.637

On the other hand, for salt extractable protein at both temperatures of −20 and 4°C, the pectin sample had the highest reaction rate constant, even larger than the control sample (at 4°C), while the reaction rate of flaxseed protein was the lowest. The reaction rate of sucrose + sorbitol + polyphosphate was higher than that of the flaxseed protein, which indicates that the remained amounts of salt extractable protein in flaxseed sample were larger than the sucrose + sorbitol + polyphosphate sample after the same storage time.

Moreover, for the sulfhydryl content, the reaction rate constants of flaxseed protein sample at both −20 and 4°C were lower than sucrose + sorbitol + polyphosphate and control sample. Results showed that modification of surimi with flaxseed protein hydrolyses had a positive effect in preserving salt extractable protein and sulfhydryl content of samples.

## 4 CONCLUSIONS

Various functional properties and protein stabilities of marine products can be deteriorated owing to long‐term storage through chilling or freezing conditions. The present study demonstrates functional characteristics (water binding capacity (%), salt extractable protein (%), sulfhydryl content) of Capoor surimi protein in the presence and absence of different groups of additives, namely, flaxseed protein hydrolysate, pectin, and commercial blends (4% sucrose + 4% sorbitol + 0.2% sodium tripolyphosphates) during various time of chilling (4°C) and freezing (−20°C). Cryoprotective effect on surimi proteins is additive type‐, temperature‐, and time‐dependent manner. Changes of the salt extractable protein and sulfhydryl content over time were fitted to the first and second‐order kinetic models for different surimi samples at temperatures of −20 and 4°C. Values of *R* ^2^ and RMSE showed that the salt extractable protein and sulfhydryl content better fit to the first‐order kinetic model at −20°C, however, second‐order reaction model has better prediction at 4°C. The reaction rate constant of flaxseed‐treated sample was the lowest in both temperatures.

## AUTHOR CONTRIBUTIONS

**Marzieh Moosavi‐Nasab Moosavi‐Nasab:** Methodology (equal); supervision (equal); writing – original draft (equal). **Leila Maghsoudi:** Methodology (equal); project administration (equal); writing – review and editing (equal). **Elahe Abedi:** Investigation (equal); writing – original draft (equal); writing – review and editing (equal). **Shahrzad Maleki:** Data curation (equal); validation (equal).

## CONFLICT OF INTEREST STATEMENT

The authors declare that they have no conflict of interest.

## ETHICAL APPROVAL

This article does not contain any studies with human participants or animals performed by any of the authors.

## CONSENT TO PARTICIPATE

The present paper has been approved by all named authors.

## CONSENT FOR PUBLICATION

The present paper, which is original, has not been published before and is not currently being considered for publication elsewhere.

## Data Availability

Research data are not shared.
